# Endoscopic Stent for Anastomotic Leakage Treatment Post Proximal Gastrectomy in Gastric Cancer: A Case Report

**DOI:** 10.7759/cureus.74941

**Published:** 2024-12-01

**Authors:** Huan Nguyen Ngoc, Ly Pham Quoc Hau

**Affiliations:** 1 Digestive Surgery, Cho Ray Hospital, Ho Chi Minh City, VNM

**Keywords:** anastomotic leakage, chemotherapy, esophagojejunostomy, fully covered self-expandable metal stent, gastric cancer surgery, gastric cardia cancer, minimal invasive surgery

## Abstract

The management of gastrointestinal anastomotic leaks post surgery is a considerable challenge, characterized by elevated morbidity and mortality, particularly in cases of esophageal-jejunal anastomotic leaks. Diverse endoscopic intervention techniques have been utilized with enhanced success. We present a case where a 57-year-old patient with Siewert type II esophageal cardia cancer underwent endoscopic deployment of a fully covered stent into a fistula resulting from anastomotic leakage, following a laparoscopic proximal gastrectomy with Roux-en-Y and double tract reconstruction. On the third day following surgery, the patient demonstrated significant respiratory distress and spat milky sputum as a result of anastomotic leakage and ensuing mediastinitis. On the 11th day after surgery, we used an esophagoscopy to insert a completely coated, self-expanding metallic stent, as the fistula was not healing. On postoperative day 54, the patient started taking oral intake and went home. After one month, we endoscopically removed the stent. Esophagoscopy following stent removal demonstrated that the fistula had resolved and the anastomotic leak had healed. This case shows that using a metal stent that can fully cover itself and expand to stop anastomotic leakage after proximal gastrectomy for stomach cancer does work.

## Introduction

According to GLOBOCAN (Global Cancer Observatory) 2022, gastric cancer is the sixth most common cancer and the fourth leading cause of cancer-related deaths worldwide [1]. Laparoscopic proximal gastrectomy with lymphadenectomy is a favorable and safe treatment option for cancer of the upper third. Due to its length and frequently insufficient patient nutrition, this surgical procedure is intricate and delicate. As a result, the occurrence of complications and severe complications prolongs treatment length and costs while significantly increasing the postoperative mortality rate.

In the past few years, there has been a lot of talk about the best way to treat esophagojejunal anastomotic fistula after proximal gastrectomy. Both surgical and conservative treatment methods are associated with increased mortality rates and prolonged hospital stays [2]. Endoscopic stent implantation for treating esophageal anastomotic leakage has shown an effectiveness rate of 70-86% in previous studies [3]. Stents have multiple benefits in the treatment of anastomotic leaks, including expedited leak closure, reduced invasiveness relative to surgical procedures, and high therapeutic effectiveness. However, stent insertion has specific disadvantages that require consideration. The occurrence of stent displacement following installation may range from 26% to 87% [3]. Partial stents pose a risk of mucosal indentation, complicating their extraction and maybe leading to additional complications. Some patients may encounter anastomotic stenosis after stent removal, requiring balloon dilation.

This report describes the successful treatment of an esophageal anastomotic fistula with a stent by gastrointestinal endoscopy.

## Case presentation

A 57-year-old male patient was transferred to the digestive surgery department with symptoms of poor appetite and a weight loss of 3 kg over the past one month. Clinical examination did not reveal any abnormalities, and all blood test results were normal. An esophagogastroduodenoscopy showed a Siewert type II tumor at the gastroesophageal junction, which is a moderately differentiated adenocarcinoma. A CT scan of the chest and abdomen showed that the cardia-esophagus wall was getting thicker in strange places, and there was a 2x3 cm tumor that was not going into the serosa (cT3NxMx). There were no signs of ascites.

The patient underwent a proximal gastrectomy, Roux en Y, and double tract gastric reconstruction through laparoscopic surgery. D2 lymph nodes were also removed. Surgery time was 360 minutes, and blood loss was 100 ml. Postoperative pathology results were moderately differentiated adenocarcinoma penetrating the serosa without lymph node metastasis (pT4aN0M0).

**Figure 1:**
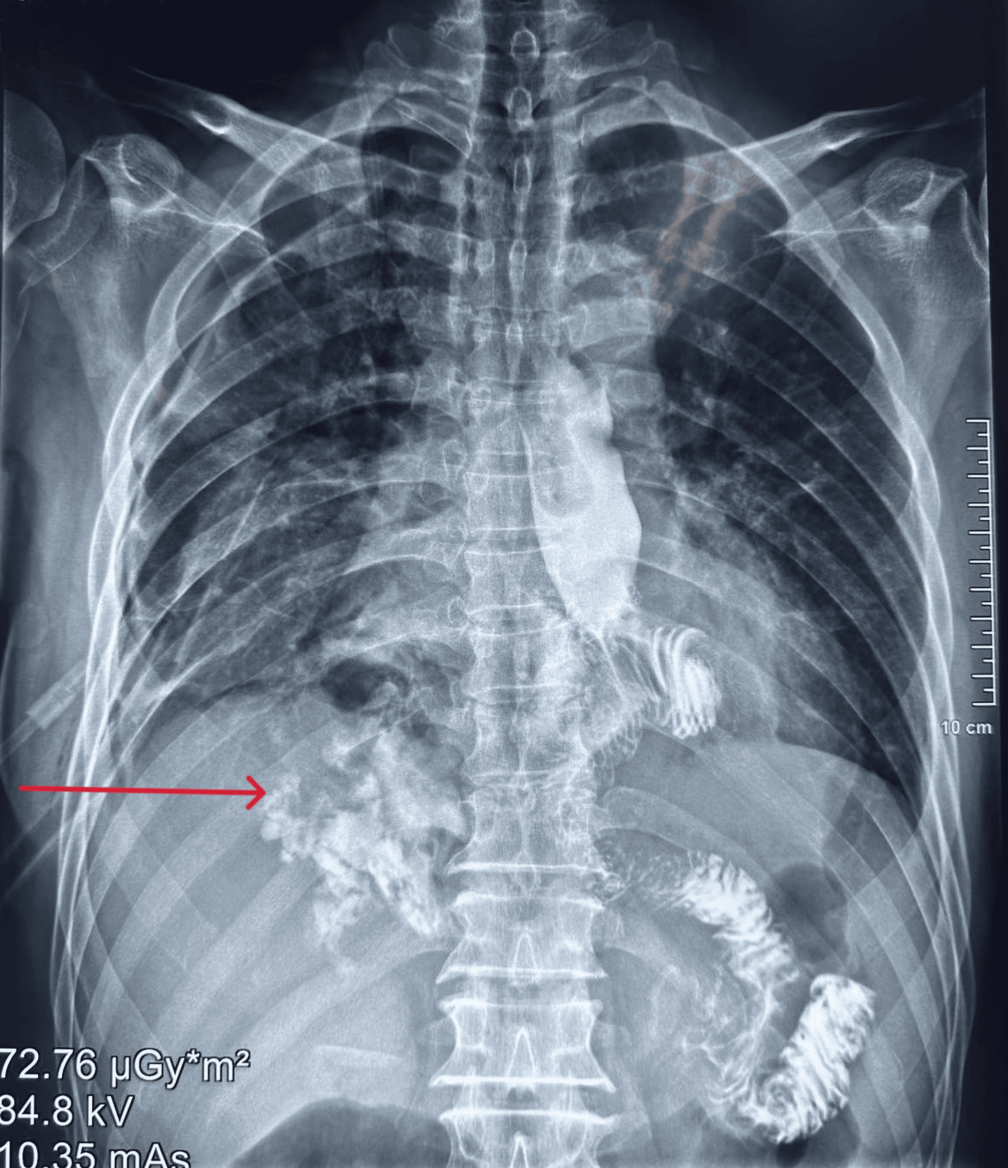
X-ray of the esophagus and stomach with contrast: note the contrast in the right chest base

**Figure 2:**
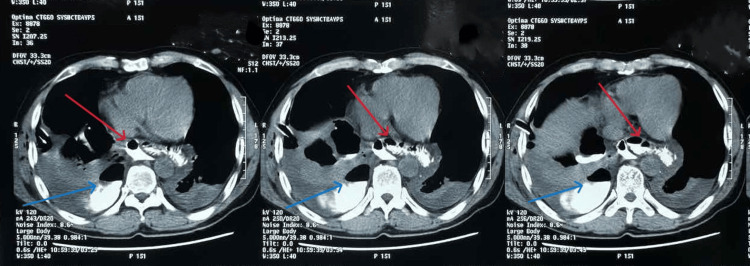
CT showing contrast dye in the esophagus leaking into the mediastinum and right pleura (red arrow) – moderate right pleural effusion (blue arrow)

On the third postoperative day, the patient experienced significant shortness of breath and coughed up white, cloudy sputum. His nasogastric tube was removed on the same day. Chest X-ray showed right lung infiltration, bilateral pleural effusion, and right lung collapse. The patient underwent right pleural drainage surgery. There was no pain in the abdomen the next day, and the shortness of breath got a little better. The surgical wound was dry and had not swollen up, and an X-ray of the esophagus and stomach with contrast was done (Figure 1). The CT scan showed that the contrast agent had leaked into the mediastinum from the esophagus and there was moderate right pleural effusion (Figure 2).

**Figure 3:**
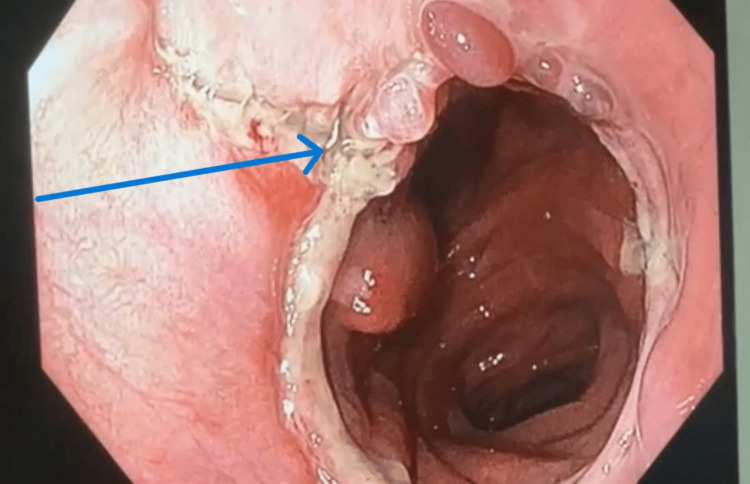
Digestive endoscopy showing fistula at the anastomosis site

**Figure 4:**
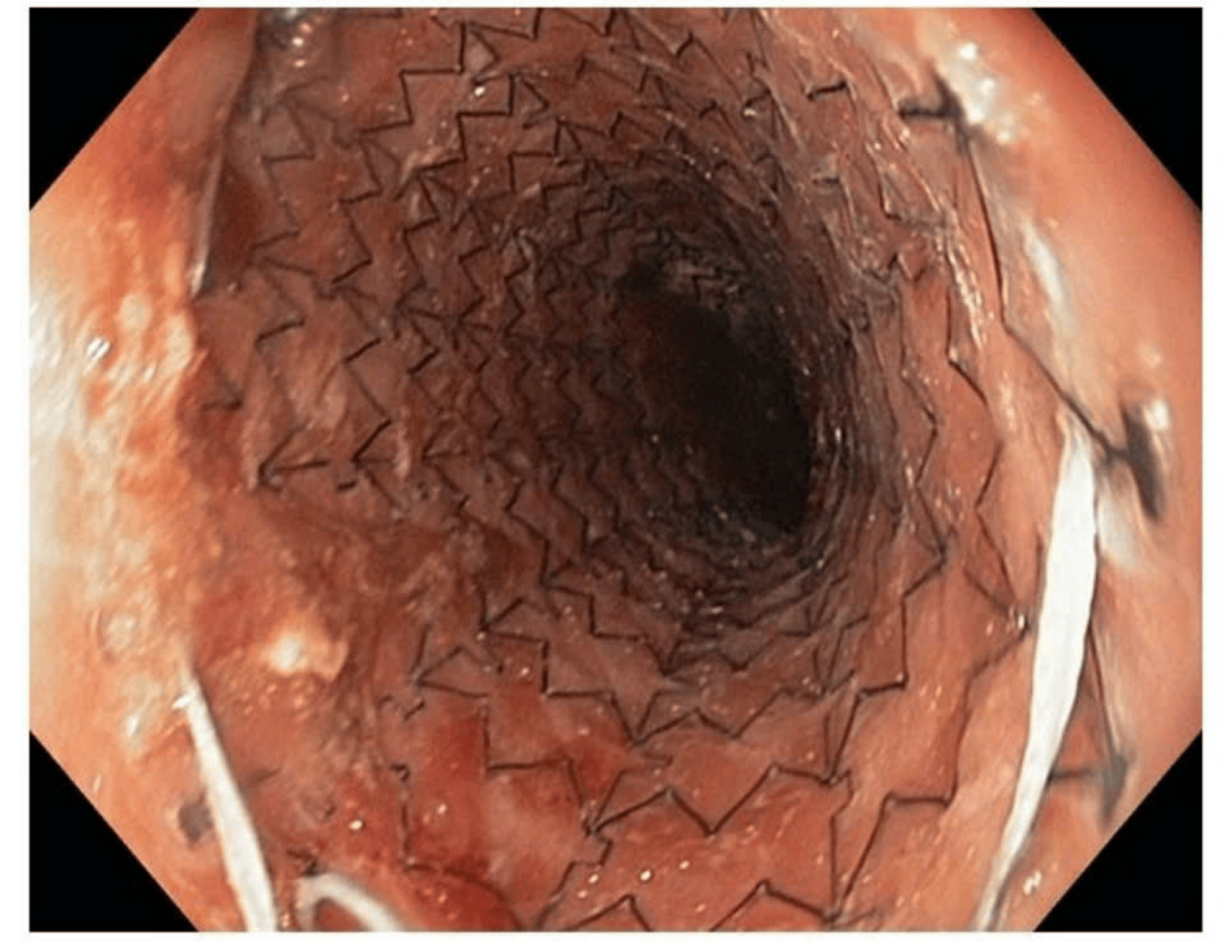
Esophageal stent

Due to the perceived elevated mortality risk during reoperation and the patient's deteriorating health status, we opted for conservative management involving endoscopic stent placement across the fistula and antibiotics. There was daily pleural irrigation in addition to the insertion of a right pleural drain. An esophagoscopy conducted on the sixth postoperative day revealed a fistula at the anastomotic location (Figure 3). An esophageal stent, specifically Choostent® (M.I. Tech, Pyeongtaek, Gyeongido, South Korea), was positioned over the fistula with the use of esophagoscopic guidance (Figure 4).

**Figure 5:**
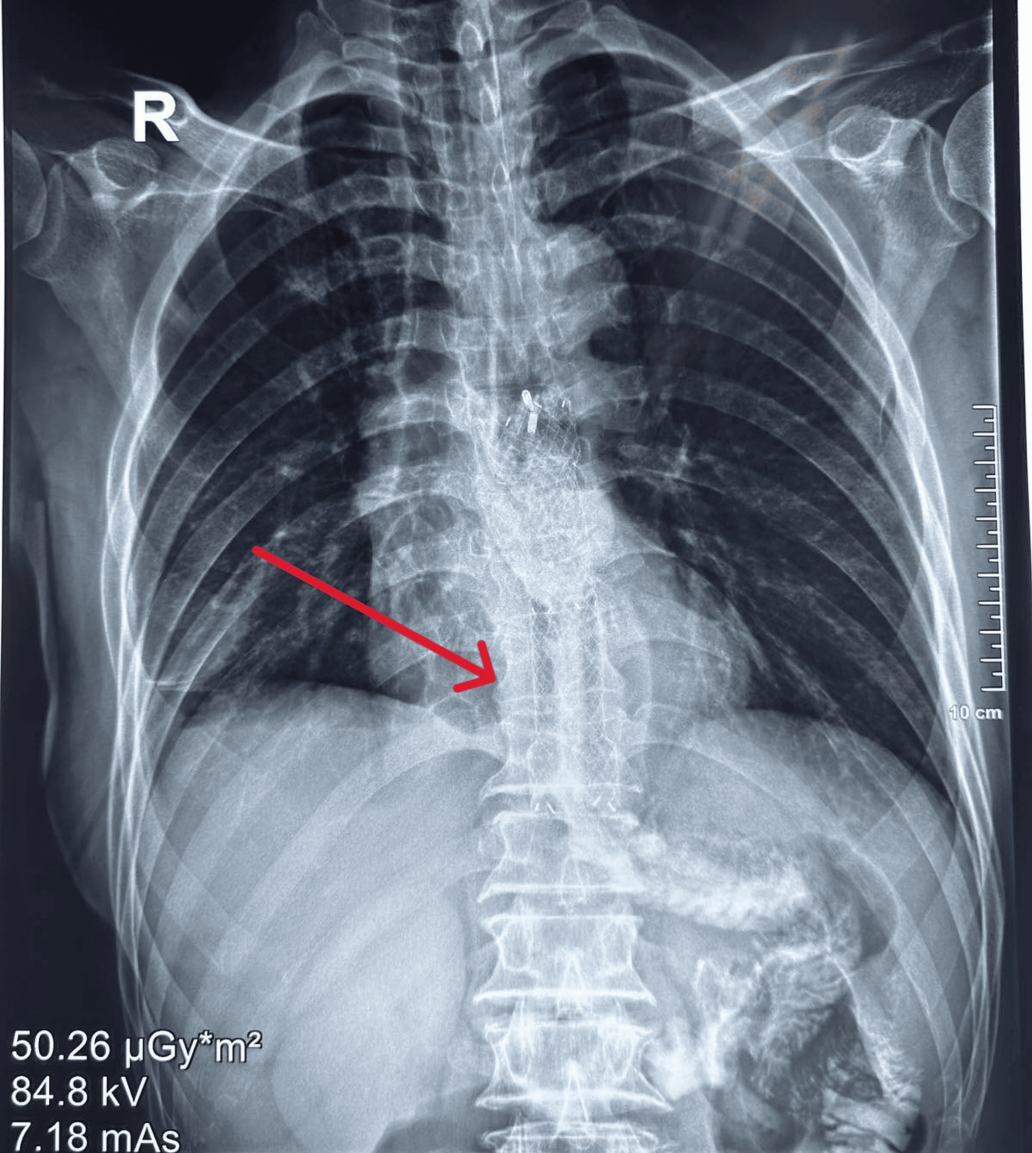
Chest X-ray after administration of water-soluble contrast medium showing no further leakage.

Postoperatively on day 11, the patient was reintroduced to oral feeding with liquid food. The pleural drainage tube was observed to be leaking 100-200 ml of turbid fluid daily, and the patient received a jejunostomy for nutritional support. After 47 days of treatment, the patient showed a clinical response. Clear fluid was draining from the pleura, and chest and esophagus X-rays with contrast dye showed no signs of a fistula (Figure 5). We resolved to extract the chest tube and permit the patient to resume oral feeding. The patient was discharged on the 54th postoperative day. We removed the stent one month after the patient was discharged from the hospital. Esophagoscopy following stent removal demonstrated that the fistula had cleared and the anastomotic leak had healed. The patient is under outpatient observation with a recurrence-free survival of six months post procedure. Despite the brevity of the follow-up time, no anastomotic stenosis has been detected.

## Discussion

Surgery is an essential element in the treatment of esophageal cardia cancer. Recent advancements in diagnostic approaches, patient selection, perioperative treatment, and surgical methods have significantly decreased surgical morbidity and mortality. The occurrence of complications related to esophagojejunal anastomosis persisted at a high level. The mechanics of staple line leakage, include a long stapling line being present, as well as the conversion of the stomach itself into a narrow, high-pressure tube due to the presence of both esophageal and pyloric sphincters [4].

Of the people who have had a laparoscopic gastrectomy, 3% experience an esophageal-jejunal anastomotic leak compared to only 2% of people who have had an open total gastrectomy [5]. Treatment options include conservative management, drainage therapy, endoscopic procedures, or revision surgery [6]. The internal stent implantation therapeutic method has initially demonstrated positive results [7]. Jung et al. did research that showed using HANAROSTENT® (M.I. Tech), to fix 14 cases of anastomotic leakage after surgery worked 85.7% of the time [8]. According to Swinnen et al., the acronym DCWR (Drain, Close, Watertightness, Remove) summarizes the principles of this procedure for resolution of leaks and perforations with success rates of 77.6% and the recorded death rate is six patients, approximately 6.81% [7].

The fluid accumulation associated with the anesthesia leak must be promptly drained. Subsequently, a self-expanding esophageal stent must be placed over the leak to ensure coverage and redirect esophageal contents from the affected region. Once the stent establishes waterproofing, the leak will spontaneously seal. After several weeks, the stent will be removed upon verification of the healing of the anastomotic leak.

Shim et al. discovered that stent placement (self-expandable metallic stent (SEMS)) was more effective than non-stent endoscopic therapies (NSET) at treating anastomotic leaks. SEMS had an 80% success rate, while NSET only had a 28.6% success rate [9]. Endoscopic stent placement has been shown to help treat esophageal-jejunal anastomotic leakage in studies conducted around the world. Despite its many advantages, stent treatment may entail specific complications. Stent migration occurs in 25-87% of stent implantation cases [3,6]. Diverse methods can be utilized to secure the stent and prevent slippage, including the application of suture anchors. The stent sticks firmly to the esophageal wall because the mucosal irritation at its ends causes tissue growth, which can cause problems during removal like bleeding, perforation, fistula formation, and abscess formation. or anastomotic stricture requiring endoscopic dilation [10,11]. Dumonceau et al. established that nitinol-coated stents exhibited a higher rate of esophageal fistula occlusion than stainless steel stents [12]. They claim that nitinol's remarkable flexibility improves the stent's adhesion to the esophageal wall, hence reducing leakage.

An endoscopic solution was chosen for our patient with an esophageal fistula because of its least invasive characteristics and superior safety profile. The stent promoted fistula closure, allowing the patient to resume eating and drinking sooner, hence limiting mediastinal infection and decreasing hospitalization length. The chosen stent was Choosent, a nitinol-coated SEMS proven to be highly effective in treating esophageal fistulas and strictures [13]. Patients who cannot control the fistula with an esophageal stent and exhibit a deteriorating infection should be referred for surgical intervention. Endoscopic stent implantation can be considered part of the therapeutic regimen, although it is not the only method employed.

## Conclusions

The progress of modern medicine has resulted in the increasing prevalence of endoscopic interventions for postoperative esophagojejunal anastomotic fistula, yielding favorable results. We report a case of successful therapy employing a SEMS.
